# Constipation induced gut microbiota dysbiosis exacerbates experimental autoimmune encephalomyelitis in C57BL/6 mice

**DOI:** 10.1186/s12967-021-02995-z

**Published:** 2021-07-23

**Authors:** Xiuli Lin, Yingying Liu, Lili Ma, Xiaomeng Ma, Liping Shen, Xueying Ma, Zhaoyu Chen, Hao Chen, Donghong Li, Zhumin Su, Xiaohong Chen

**Affiliations:** Department of Neurology and Multiple Sclerosis Research Center, The Third Affiliated Hospital of Sun Yat-sen University, Guangzhou, Guangdong China

**Keywords:** Constipation, Experimental autoimmune encephalomyelitis, Gut microbiota dysbiosis, Treg/Th17 and Treg17/Teff17 imbalance, Cytokine-balance disturbance

## Abstract

**Background:**

Constipation is a common gastrointestinal dysfunction which has a potential impact on people's immune state and their quality of life. Here we investigated the effects of constipation on experimental autoimmune encephalomyelitis (EAE), an animal model of multiple sclerosis (MS).

**Methods:**

Constipation was induced by loperamide in female C57BL/6 mice. The alternations of gut microbiota, permeability of intestinal barrier and blood–brain barrier, and histopathology of colon were assessed after constipation induction. EAE was induced in the constipation mice. Fecal microbiota transplantation (FMT) was performed from constipation mice into microbiota-depleted mice. Clinical scores, histopathology of inflammation and demyelination, Treg/Th17 and Treg17/Teff17 imbalance both in the peripheral lymphatic organs and central nervous system, cytokines include TGF-β, GM-CSF, IL-10, IL-17A, IL-17F, IL-21, IL-22, and IL-23 in serum were assessed in different groups.

**Results:**

Compared with the vehicle group, the constipation mice showed gut microbiota dysbiosis, colon inflammation and injury, and increased permeability of intestinal barrier and blood–brain barrier. We found that the clinical and pathological scores of the constipation EAE mice were severer than that of the EAE mice. Compared with the EAE mice, the constipation EAE mice showed reduced percentage of Treg and Treg17 cells, increased percentage of Th17 and Teff17 cells, and decreased ratio of Treg/Th17 and Treg17/Teff17 in the spleen, inguinal lymph nodes, brain, and spinal cord. Moreover, the serum levels of TGF-β, IL-10, and IL-21 were decreased while the GM-CSF, IL-17A, IL-17F, IL-22, and IL-23 were increased in the constipation EAE mice. In addition, these pathological processes could be transferred via their gut microbiota.

**Conclusions:**

Our results verified that constipation induced gut microbiota dysbiosis exacerbated EAE via aggravating Treg/Th17 and Treg17/Teff17 imbalance and cytokines disturbance in C57BL/6 mice.

**Supplementary Information:**

The online version contains supplementary material available at 10.1186/s12967-021-02995-z.

## Background

Constipation is a common clinical symptom of gastrointestinal dysfunction worldwide. It is characterized by difficult or infrequent passage of stool, hardness of stool, and/or a feeling of incomplete evacuation [1, 2]. Due to the stressful lifestyle, the prevalence of constipation is estimated to be as high as 20% in some populations including Asia [3–5]. It affects the quality of people’s life among all ages and genders [3–5]. Growing evidences have indicated that constipation can lead to gut microbiota dysbiosis [1]. Gut microbiota plays an important role in regulating T cells differentiation and cytokines secretion. The alterations in gut microbiota have been implicated in the pathogenesis of both intestinal and non-intestinal autoimmune diseases such as ulcerative colitis and Crohn’s disease, rheumatoid arthritis and multiple sclerosis (MS) [6–8].

MS is a chronic inflammatory, demyelinating, and degeneration autoimmune disease in the central nervous system (CNS) [9]. MS could be triggered by both genetic factors and environmental exposures. Its incidence rate has increased with the changed and stressful lifestyle [10]. Majority of MS patients exhibit constipation which could not be simply explained by the presence of CNS lesions, and constipation could precede the clinical symptoms of CNS in some MS patients [11, 12]. At present, the effects of constipation on MS and the related mechanism remain unclear.

Regulatory T (Treg) cells and T helper (Th) 17 cells are two particular T cell subsets with plasticity playing important roles in the immunity of the intestine and CNS [13–15]. Treg cells play a fundamental role in maintaining immune homeostasis and inhibiting autoimmunity in MS/EAE by suppressing the activation of other immune cell types [13–15]. Th17 cells are thought to promote CNS autoimmunity and strongly associated with disease activity and CNS dysfunction [13, 14]. Treg cells and Th17 cells can convert into each other, and the Treg/Th17 balance is vital to the immunity of intestinal mucosal barriers and the inflammatory responses in MS [14, 16, 17]. Recent evidences have reported that Th17 cells can differentiate into either protective or pathogenic cells under the surrounding microenvironment [18]. Th17 cells that induced by interleukin (IL)-6 and transforming growth factor β (TGF-β) can convert into regulatory Th17 (Treg17) cells, which are protective cells that negatively regulate inflammation response [18]. Th17 cells that induced by IL-6 and IL-23 are termed as the effector Th17 (Teff17) cells. They are pathogenic cells enhancing inflammation response [18]. It has been reported that Treg17/Teff17 imbalance plays an essential role in the development of pulmonary vasculitis, lupus nephritis, and glomerulonephritis [19, 20]. As T cells abundantly reside in the intestinal lamina propria [16], it is worthy to explore whether the alteration of intestinal microbiota microenvironment that induced by constipation has the effects on the balance of Treg/Th17 and Treg17/Teff17 in experimental autoimmune encephalomyelitis (EAE), a classical model of MS.

In this study, EAE was induced in the loperamide-induced constipation mice. Fecal microbiota transplantation (FMT) was performed from constipation mice into microbiota-depleted mice to verify whether constipation relevant pathological changes could be transferred via their gut microbiota. Our results suggested that constipation induced gut microbiota dysbiosis exacerbated EAE through aggravating Treg/Th17 and Treg17/Teff17 imbalance and cytokines disturbance. And these pathological processes could be transferred via their gut microbiota by FMT. As constipation is one of the most important symptoms related to stressful lifestyle, this study implies the potential of lifestyle modulation in the alleviation of MS.

## Material and methods

### Animals and regents

Four- to five-week-old female C57BL/6 mice were obtained from the Experimental Animal Center of Guangdong (Guangzhou, China). Experiments were carried out according to the National Institutes of Health Guide for the Care and Use of Laboratory Animals (8th edition, 2011) [21] and approved by the Bioethics Committee of South China Agricultural University (Approval ID: 2018D066). Mice were allowed to acclimatize to the laboratory for 1 week prior to the study. Loperamide hydrochloride was purchased from MedChemExpress (New Jersey, USA). Amphotericin B, vancomycin hydrochloride, ampicillin, metronidazole, neomycin sulfate, 150% (w/v) barium sulfate (BaSO_4_) were purchased from Sangon Biotech (Shanghai, China). Fluorescein isothiocyanate-dextran 4000 (FITC-D4000), Evans blue (EB), and complete Freund’s adjuvant (CFA) were purchased from Sigma-Aldrich (St. Louis, USA). A myelin oligodendrocyte glycoprotein (MOG) peptide _35–55_ (MEVGWYRSPFSRVVHLYRN GK) was synthesized by CL, Bio-Scientific Co., Ltd (Xi’an, China). Amino acid sequences were confirmed by amino acid analysis and mass spectrometry. The purity of the peptide was greater than 95%. *Mycobacterium tuberculosis* H37RA was purchased from Difco (Detroit, MI, USA). Pertussis toxin (PTX) was purchased from List Biological Lab (Epsom, England). The TGF-β enzyme-linked immunosorbent assay (ELISA) kits and ProcartaPlex™ multiplex immunoassay kit for granulocyte–macrophage colony-stimulating factor (GM-CSF), IL-10, IL-17A, IL-17F, IL-21, IL-22, IL-23 were purchased from Thermo Fisher Scientific (Massachusetts, USA). Anti-CD4 FITC-conjugated, anti-IL-17A PE-conjugated, anti-Foxp3 PE-Cy7-conjugated antibodies were purchased from Thermo Fisher Scientific (Massachusetts, USA).

### Experimental design

Four- to five-week-old female C57BL/6 mice were randomly allocated into three groups: (i) vehicle mice accepted saline solution treatment; (ii) constipation mice accepted loperamide treatment; and (iii) microbiota-depleted mice accepted antibiotic cocktail treatment. Freshly extruded stools were collected right after the constipation induction or microbiota depletion for fecal parameters measurement and 16S rRNA sequencing analysis. FMT was performed from constipation mice into microbiota-depleted mice (FMT mice). After the experiments mentioned above, mice accepted EAE induction according to the protocol described below or phosphate-buffered saline (PBS) injection as control. The four groups were described as vehicle mice, EAE mice, constipation EAE mice, and FMT EAE mice. Every experiment was repeated three times.

### Induction of experimental constipation

Loperamide hydrochloride was dissolved in the sterile saline solution to a final concentration of 2.4 mg/ml. Constipation was induced in mice by oral gavage at a dose of 9.6 mg/kg twice a day for 2 weeks [22]. The vehicle mice accepted equivalent saline solution by oral gavage alone.

### Fecal parameters measurement

Vehicle mice and constipation mice were fed freely and observed in a single cage immediately after the constipation induction. The pellets were collected and counted every 10 min for 2 h. The pellets were weight and dried at 70 ℃ for 24 h in a laboratory dry-oven. The content of fecal water was calculated according to the equation (wet weight – dry weight)/wet weight of fecal pellets × 100% [22]. Gastrointestinal transit was evaluated as previously described [22]. Briefly, after fasting 12 h with free access to water, the mice were fed with 150% (w/v) BaSO_4_ by oral gavage. After that, the fecal pellets were monitored every 10 min for the presence of the first white pellet. The time when the first white pellet ejected was recorded.

### 16S rRNA gene sequencing

Freshly extruded stools were collected right after the constipation induction or intestinal microbiota depletion for fecal parameters measurement and 16S rRNA sequencing analysis at BGI Co. (Shenzhen, China) according to our previously published study [23]. Briefly, DNA was extracted using QuickGene DNA tissue kit from Kurabo Company (Neyagawa, Japan) and next used for PCR amplification and sequencing of the V3 and V4 region of bacterial 16S rRNA genes with Illumina MiSeq technology.

Gene catalog construction, taxonomic annotation, and abundance calculation were performed according to the published study [24]. Alpha-diversity was calculated according to the indexes of observed species, Chao, Ace, Shannon, and Simpson. Partial least squares discrimination analysis (PLS-DA) was performed based on operational taxonomic units (OTU) abundance information using QIIME (Quantitative Insights into Microbial Ecology, version1.8.0). The differential microbial flora biomarkers among groups were performed using linear discriminant analysis (LDA) effect size (LEfSe) analysis [24].

### Transmission electron microscopy

After the induction of constipation, the distal colon of vehicle mice and constipation mice were isolated and fixed at 4 °C in 4% glutaraldehyde/4% paraformaldehyde (PFA) for transmission electron microscopy (TEM) according to the protocol [9]. Specimens were then rinsed with phosphoric acid, dehydrated with acetone, immersed, embedded in EPON812, and cut into Ultrathin sections (60 nm) using an ultratome. Grids were observed using Tecnai G2 Spirit TWIN TEM (FEI, Oregon USA).

### Assessment of the intestinal barrier and blood–brain barrier

The FITC-D4000 test was performed to assess the intestinal barrier function [25]. Mice were given FITC-D4000 at a dose of 600 mg/kg body weight by oral gavage. Then the mice fasted with free access to water in the metabolic cages for 1 h. After the experiment, blood from different groups were collected by cardiocentesis and heparinized, and then centrifuged at the condition: 10 min, 12,000*g*, 4 ℃. The collected plasm was protected from light and stored at – 80 ℃ immediately for the FITC-D4000 test. FITC-D4000 was diluted with plasma from untreated mice (blank) as standard (range 0.312–50 μg/ml). An amount of 100 μl of plasma from different groups and standards were transferred to black 96-well microplates for analysis of FITC-D4000 concentration using SpectraMax M5 according to the guidelines of Molecular devices (Silicon valley, USA).

Blood–brain barrier (BBB) permeability was assessed with EB perfusion as previously described [26]. Mice were intravenously injected with 0.4% EB at a dose of 200 mg/kg and circulated for 30 min. Then mice were fixed by cardiac perfusion with PBS and 4% (w/v) PFA. Brains were obtained and tissue cryo-sections were analyzed by fluorescence microscopy.

### Intestinal microbiota depletion and fecal microbiota transplantation

Microbiota-depleted mice were induced with an antibiotic cocktail consisted of amphotericin B (0.1 mg/ml), vancomycin hydrochloride (5 mg/ml), ampicillin (10 mg/ml), metronidazole (10 mg/ml) and neomycin sulfate (10 mg/ml) by oral gavage at a dose of 10 ml/kg twice per day for 2 weeks [24]. FMT was performed immediately after the intestinal microbiota depletion. Fresh stools were collected from constipation mice immediately after constipation induction and homogenized with a sterile saline solution at the concentration of 5 mg/ml under anaerobic conditions (in the above of carbon dioxide ice). Supernatants were collected after 5 min standing and given to microbiota-depleted mice twice per day for 3 weeks by oral gavage (200 μl per mouse) [24].

### EAE induction and assessment

EAE was induced as previously described immediately after the fecal collection [27]. Briefly, mice immunized subcutaneously in the flanks of 200 μg MOG_35–55_ peptide emulsified in CFA including 500 μg *mycobacterium tuberculosis* H37RA on day 0 and day 7. Immediately thereafter and on day 2, the mice received an intraperitoneal injection of 300 ng PTX in 100 μl PBS. Mice were scored daily and blindly by two researchers individually using a clinical scoring system ranging from 0 to 5 as follows: grade 5, death; grade 4.5, near death, moribund; grade 4, complete paralysis of two limbs; grade 3, complete paralysis of a single limb; grade 2.5, partial limb paralysis and ataxia; grade 2, dysfunctional gait with limp tail and ataxia; and grade 1, dysfunctional gait with tail tonicity or limp tail [28].

### Histopathology

The distal colon of vehicle mice and constipation mice were isolated for further pathological studies. On the 25st day post-immunization (p.i.) to initiate EAE, lumbar spinal cords were isolated from the EAE mice, constipation EAE mice, and FMT EAE mice for pathological studies. Mice from different groups were fixed by cardiac perfusion with 4% (w/v) PFA, and the distal colon and the spinal cord were obtained and embedded in paraffin. Sections of the distal colon were stained with hematoxylin and eosin (HE) to reveal adrenal hyperplasia. The spinal cord samples were stained with HE and luxol fast blue (LFB) to evaluate inflammatory cell infiltration and demyelination, respectively. The severity of inflammatory cell infiltration was scored as followed: 0, no inflammatory cells; 1, a few scattered inflammatory cells; 2, organization of inflammatory infiltrates around blood vessels; 3, extensive perivascular cuffing with extension into adjacent parenchyma, or parenchymal infiltration without obvious cuffing [29]. The demyelination was scored as followed: 1, traces of subpial demyelination; 2, marked subpial and perivascular demyelination; 3, confluent perivascular or subpial demyelination; 4, massive perivascular and subpial demyelination involving one half of the spinal cord with presence of cellular infiltrates into CNS parenchyma; 5, extensive perivascular and subpial demyelination involving the whole cord section with presence of cellular infiltration into CNS parenchyma [30].

### Flow cytometry

The spleen, inguinal lymph nodes (ILNs), brain, and spinal cord from different treated mice were collected for the flow cytometry analysis. CNS-infiltrating mononuclear cells and lymphocytes isolated from these tissues were isolated as described previously [31]. Cells were fixed and permeabilized [32], followed by fluorophore-conjugated intracellular cytokine-specific antibody staining and nuclear transcription factor-specific antibody staining. Samples were measured by a CytoFLEX and then analyzed by FlowJo (Tree Star, Ashland, OR).

### Analysis of cytokines production

The serum from different treated mice were aseptically harvested on 25st days p.i. and stored at − 80 °C immediately. The concentration of TGF-β was determined by ELISA according to the guidelines of the manufacturer’s protocol. The production of GM-CSF, IL-10, IL-17A, IL-17F, IL-21, IL-22, and IL-23 were measured using a ProcartaPlex™ multiplex immunoassay according to the guidelines of the manufacturer’s protocol.

### Statistical analysis

Data were expressed as mean ± standard error of the mean (SEM). The statistical analysis was performed using SPSS 20.0 (SPSS, Inc., Chicago, IL, USA). Bioinformatics data were analyzed using Wilcoxon rank-sum test. Differences between clinical scores and histopathology scores were estimated by Kruskal–Wallis test. One-way ANOVA or Student’s t-test was performed for the rest comparison between groups. When ANOVA showed significant differences, pair-wise comparisons between means were tested by Bonferroni post-hoc test or Dunnett’s tests. Values of *p* < 0.05 were considered statistically significant.

## Results

### Constipation induced gut microbiota dysbiosis in mice

**Fig. 1:**
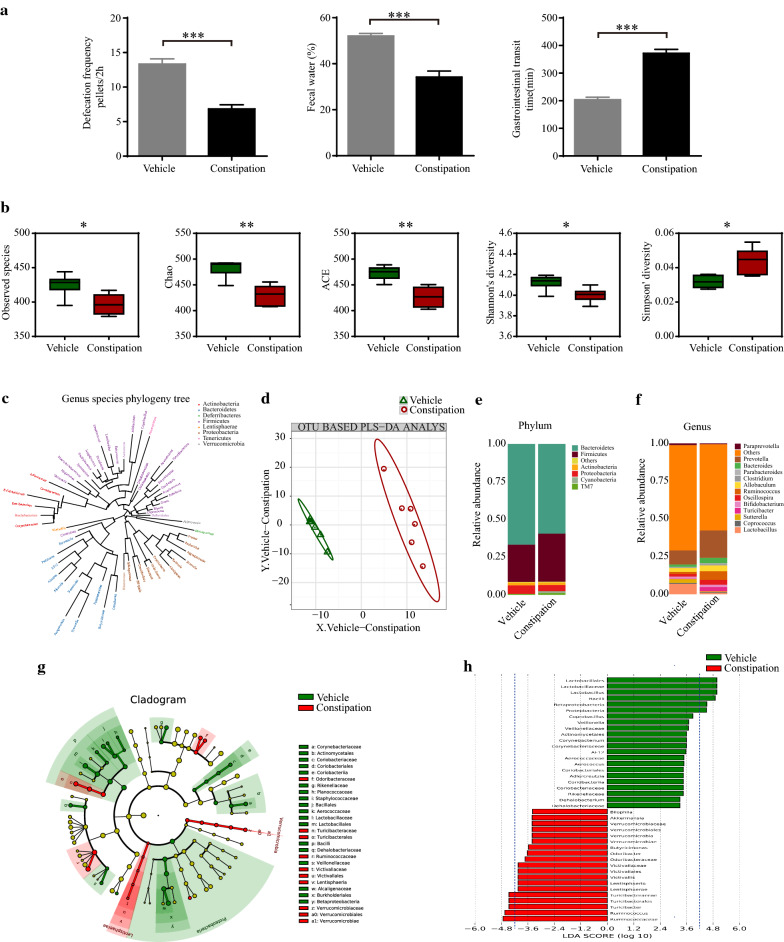
Constipation induced gut microbiota dysbiosis in mice. Constipation was induced by loperamide in four- to five -week-old female C57BL/6 mice. Fecal parameters including defecation frequency pellets/2 h, fecal water (%), and gastrointestinal transit time were measured right after the induction of constipation (**a**). Fresh extruded stools were collected right after the constipation induction for 16 s rRNA sequencing analysis. The indexes of observed species, Chao, Ace, Shannon and Simpson in the vehicle mice and constipation mice (**b**). Genus species phylogeny tree (**c**). PLS-DA analysis based on OTU abundance information (**d**). Relative abundances of the gut microbiota at phylum level (**e**) and genus level (**f**). Cladogram using LEfSe analysis indicated the phylogenetic distribution of gut microbiota (**g**). LDA scores showed the significant bacterial differences between the vehicle mice and constipation mice (**h**). N = 6 in each group. Data are displayed as mean ± SEM. *p < 0.05, **p < 0.01, ***p < 0.001

Constipation was induced by loperamide in four- to five-week-old female C57BL/6 mice. The fecal parameters of the constipation mice presented less defecation pellets (6.83 ± 0.60 vs. 13.33 ± 0.76, *p* < 0.001), fewer fecal water percentage (34.21 ± 2.64 vs. 52.07 ± 1.08, *p* < 0.001), and increased gastrointestinal transit time (371.67 ± 14.47 vs. 203.33 ± 9.19, *p* < 0.001) compared with vehicle mice (Fig. 1a).

Gut microbiota was analyzed by 16S rRNA gene sequencing. We found that constipation mice exhibited significantly reduced abundance and diversity of gut microbiota showing as decreased observed species (396.67 ± 5.96 vs. 428.50 ± 7.46, *p* < 0.05), indexes of Chao (430.16 ± 7.77 vs. 483.61 ± 7.27, *p* < 0.01), ACE (426.47 ± 8.49 vs. 473.11 ± 5.46, *p* < 0.01), Shannon (4.00 ± 0.03 vs. 4.13 ± 0.03, *p* < 0.05), and increased indexes of Simpson (0.05 ± 0.00 vs. 0.03 ± 0.00, *p* < 0.05) (Fig. 1b). Genus species phylogeny tree revealed the relationship between intestinal flora compositions in mice (Fig. 1c). By using PLS-DA, a distinct clustering gut microbiota composition was presented between the constipation mice and vehicle mice (Fig. 1d). In the phylum level, the relative abundance of *Firmicutes* was increased while the relative abundance of *Bacteroidetes* was decreased (that is the elevated *Firmicutes*/*Bacteroidetes* ratio, F/B ratio) in constipation mice (Fig. 1e). In the genus level, we also observed that the *Prevotella*, *Ruminococcus*, and *Turicibacter* were increased while the *Lactobacillus* was decreased in constipation mice (Fig. 1f). LEfSe analysis was performed to identify the differential microbial biomarkers between the two groups (Fig. 1g, h). The LDA score showed that constipation greatly increased the levels of *Turicibacter* and *Ruminococcus* while decreased the levels of *Lactobacillus* compared to vehicle mice.

Verified by 16S rRNA gene sequencing, antibiotic cocktail treatment significantly depleted the gut microbiota in microbiota-depleted mice (Additional file 1: Fig. S1).

### Constipation induced colon inflammation and injury, increased the permeability of intestinal barrier and BBB in mice

**Fig. 2:**
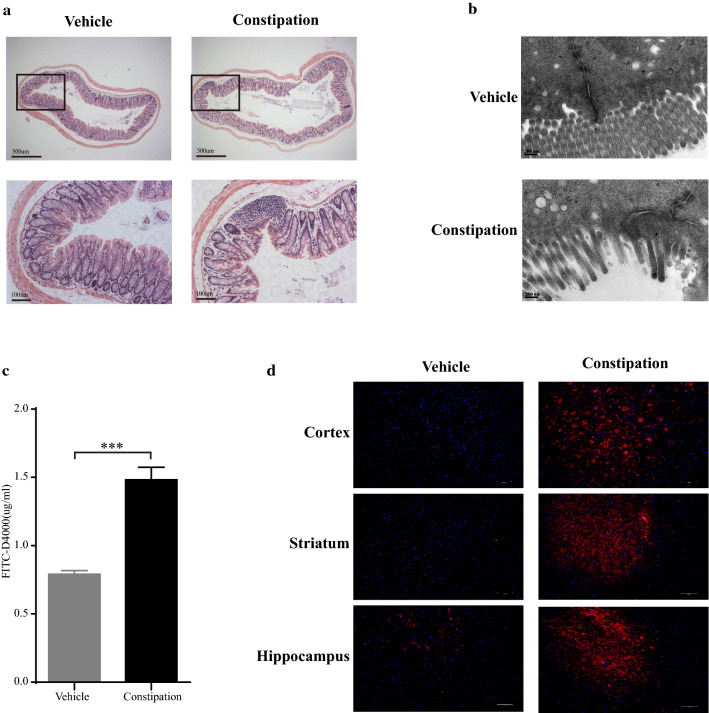
Constipation induced colon inflammation and injury, increased permeability of intestinal barrier and BBB in mice. After the constipation induction, distal colon was isolated and performed HE staining (× 50, scale bars = 500 μm; × 200, scale bars = 100 μm) (**a**) and TEM (× 42,000, scale bars = 200 nm) (**b**) for the pathological assessment. Mice were given FITC-D4000 at a dose of 600 mg/kg by oral gavage and the plasm was collected after 1 h to assess the intestinal permeability. The FITC-D4000 concentration in the plasm from different groups (**c**). Mice were intravenously injected with 0.4% EB at a dose of 200 mg/kg and circulated for 30 min. Cortex, striatum and hippocampus were obtained and tissue cryo-sections were analyzed by fluorescence microscopy for BBB permeability assessment (**d**). N = 6 in each group. Data are displayed as mean ± SEM. *p < 0.05, **p < 0.01, ***p < 0.001

HE staining and TEM were performed to evaluate the pathological changes of distal colon in constipation mice. As shown in Fig. 2a, a clear inflammatory cell infiltration with decreased amount and irregularly arranged microvilli were observed in the distal colon of constipation mice. The results in TEM were consistent with HE staining. Moreover, the structure of tight junctions was disrupted and the electron-dense materials were clearly reduced in constipation mice (Fig. 2b).

FITC-D4000 test was performed to assess the intestinal permeability. As shown in Fig. 2c, the concentration of FITC-D4000 significantly increased in constipation mice compared with the vehicle mice (1.48 ± 0.09 vs. 0.79 ± 0.03, *p* < 0.001). Then we used EB perfusion to analyze the BBB permeability. Our results showed that the presence of EB dye (bright red) existed in both blood vessels and the brain parenchyma of constipation mice, while only existed in the blood vessels of vehicle mice (Fig. 2d). These findings indicated that both the intestinal barrier and BBB were damaged in constipation mice.

### Constipation induced gut microbiota dysbiosis aggravated the severity of EAE

**Fig. 3:**
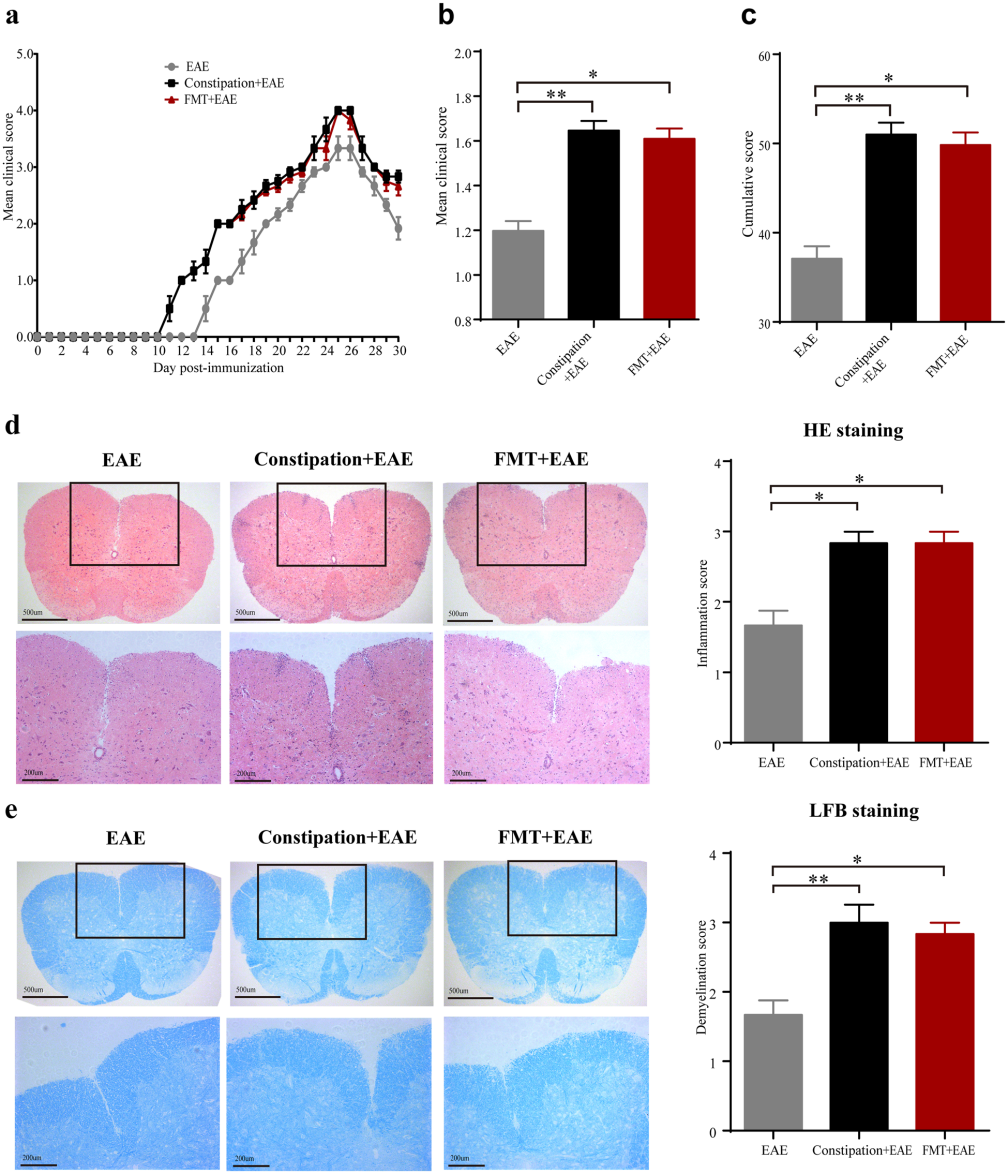
Constipation induced gut microbiota dysbiosis aggravated the severity of EAE. MOG_35–55_ peptide was adopted for the induction of EAE in female C57BL/6 mice immediately after the fecal collection. Daily clinical scores (**a**), mean clinical scores (**b**), and cumulative scores (**c**) of each group mice. On the 25st day p.i. to initiate EAE, lumbar spinal cords were isolated from the mice for pathological studies. The lumbar spinal cords were stained with HE and LFB to evaluate inflammatory cell infiltration and demyelination, respectively. Representative sections (× 50, scale bars = 500 μm; × 100, scale bars = 200 μm) and statistical analysis of the histopathological degree of the lumbar spinal cords of mice from three different groups are shown (**d**, **e**). N = 6 in each group. Data are displayed as mean ± SEM. *p < 0.05, **p < 0.01, ***p < 0.001

EAE was induced in constipation mice and FMT mice. The disease onset day indicated the day when an individual mouse showed the first symptom. The mean clinical score represented the average score of each mouse during the experiment. The overall disease burden of each mouse was presented as cumulative score. We found both constipation EAE mice and FMT EAE mice showed earlier onset of disease (11.50 ± 0.22 vs. 14.5 ± 0.22, *p* < 0.01 for constipation EAE mice; 11.50 ± 0.22 vs. 14.5 ± 0.22, *p* < 0.01 for FMT EAE mice) than EAE mice (Fig. 3a). Compared to EAE mice, both constipation EAE mice and FMT EAE mice showed higher mean clinical scores (1.65 ± 0.04 vs. 1.20 ± 0.04, *p* < 0.01 for constipation EAE mice; 1.61 ± 0.05 vs. 1.20 ± 0.04, *p* < 0.05 for FMT EAE mice) and cumulative score (51.00 ± 1.34 vs. 37.08 ± 1.40, *p* < 0.01 for constipation EAE mice; 49.83 ± 1.40 vs. 37.08 ± 1.40, *p* < 0.05 for FMT EAE mice) (Fig. 3b, c).

The HE and LFB staining of lumbar spinal cord showed more inflammatory cell infiltration (2.83 ± 0.17 vs. 1.67 ± 0.21, *p* < 0.05 for constipation EAE mice; 2.83 ± 0.17 vs. 1.67 ± 0.21, *p* < 0.05 for FMT EAE mice) and severer demyelination (3.00 ± 0.26 vs. 1.67 ± 0.21, *p* < 0.01 for constipation EAE mice; 2.83 ± 0.17 vs. 1.67 ± 0.21, *p* < 0.05 for FMT EAE mice) in constipation EAE mice and FMT EAE mice compared to EAE mice (Fig. 3d, e).

### Constipation induced gut microbiota dysbiosis aggravated Treg/Th17 and Treg17/Teff17 imbalance in EAE mice

**Fig. 4:**
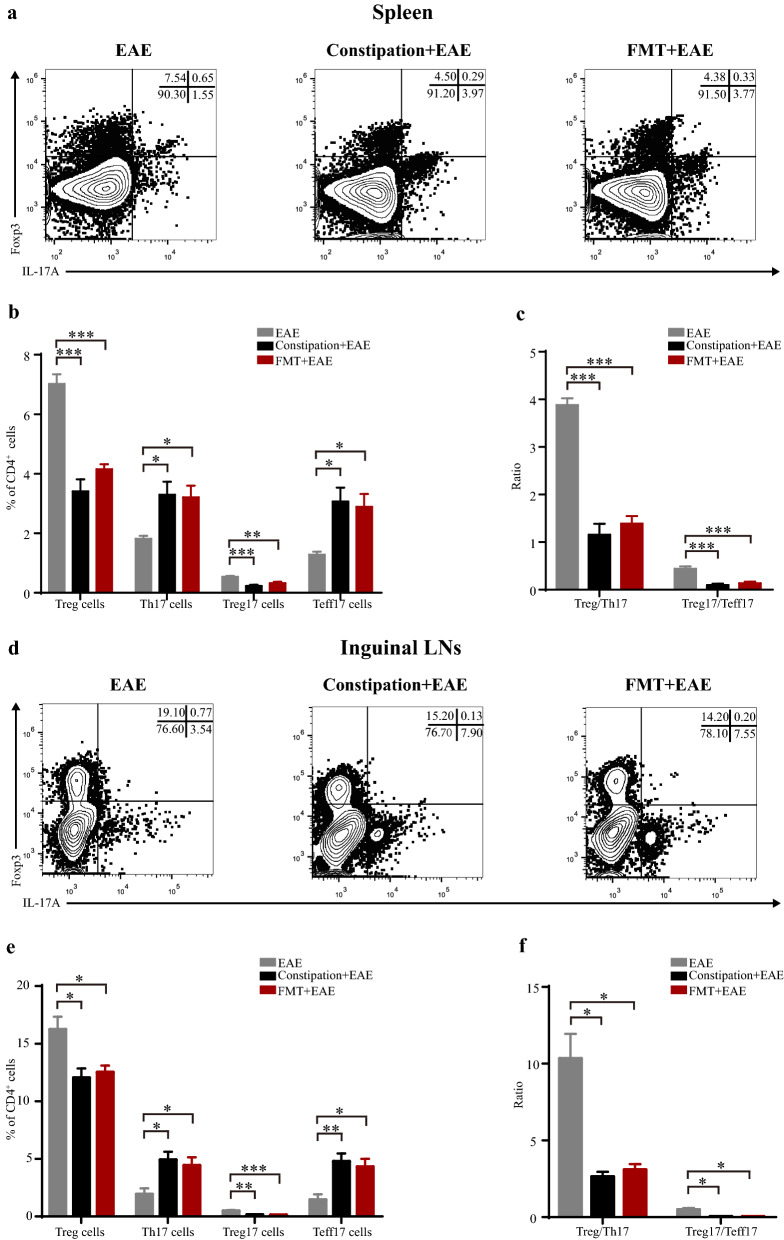
Constipation induced gut microbiota dysbiosis aggravated Treg/Th17 and Treg17/Teff17 imbalance in peripheral lymphoid organs of EAE mice. Lymphocytes were harvested from the spleens and ILNs of all mice on 25^st^ day p.i. and subsequently analyzed by flow cytometry. Mononuclear cells were first gated for lymphocytes followed by the gating of CD4^+^ T cells, and then analyzed for the percentage of Treg (CD4^+^Foxp3^+^) cells, Th17 (CD4^+^IL-17A^+^) cells, Treg17 (CD4^+^IL-17A^+^ Foxp3^+^) cells and Teff17 (CD4^+^IL-17A^+^ Foxp3^−^) cells. Representative flow data showed percentage of Treg, Th17, Treg17 and Teff17 cells in the spleens and ILNs gated on CD4 positive cells of three groups (**a**, **d**). Quantification of the percentage of Treg, Th17, Treg17, Teff17 cells in the spleens and ILNs from three groups (**b**, **e**). Quantification of ratio of Treg/Th17 and Treg17/Teff17 in the spleens and ILNs from three groups (**c**, **f**). N = 6 in each group. Data are displayed as mean ± SEM. *p < 0.05, **p < 0.01, ***p < 0.001

**Fig. 5:**
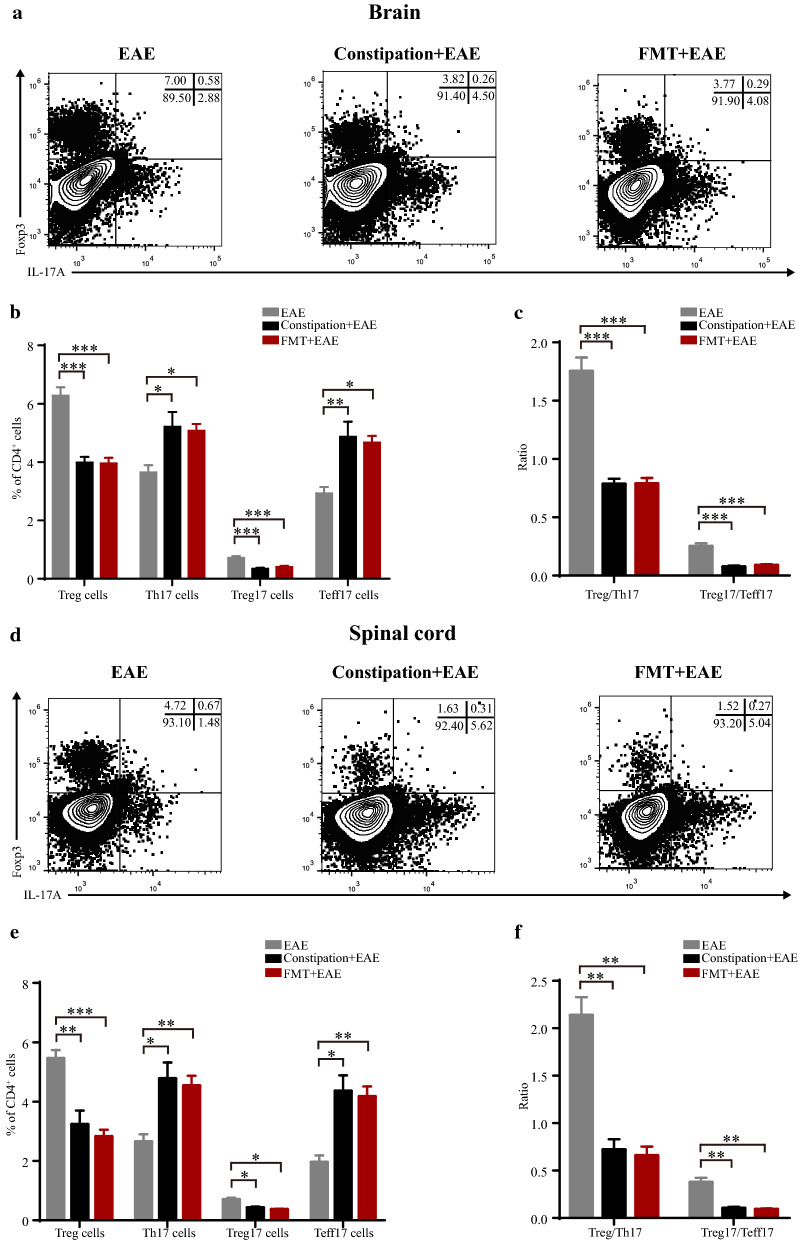
Constipation induced gut microbiota dysbiosis aggravated Treg/Th17 and Treg17/Teff17 imbalance in CNS of EAE mice. Lymphocytes were harvested from the brains and spinal cords of all mice on 25^st^ day p.i. and subsequently analyzed by flow cytometry. Mononuclear cells were first gated for lymphocytes followed by the gating of CD4^+^ T cells, and then analyzed for the percentage of Treg (CD4^+^Foxp3^+^) cells, Th17 (CD4^+^IL-17A^+^) cells, Treg17 (CD4^+^IL-17A^+^ Foxp3^+^) cells and Teff17 (CD4^+^IL-17A^+^ Foxp3^−^) cells. Representative flow data showed percentage of Treg, Th17, Treg17 and Teff17 cells in the brains and spinal cords gated on CD4 positive cells of three groups (**a**, **d**). Quantification of the percentage of Treg, Th17, Treg17, Teff17 cells in the brains and spinal cords from three groups (**b**, **e**). Quantification of ratio of Treg/Th17 and Treg17/Teff17 in the brains and spinal cords from three groups (**c**, **f**). N = 6 in each group. Data are displayed as mean ± SEM. *p < 0.05, **p < 0.01, ***p < 0.001

Th17 cells and Treg cells are two particular T cell subsets with plasticity playing important roles in the immunity of the intestine and CNS [15–17]. We further analyzed the percentage of Treg, Th17, Treg17, and Teff17 cells by flow cytometry in the EAE mice, constipation EAE mice, and FMT EAE mice. We found that the percentage of Treg and Treg17 cells were reduced, while Th17 and Teff17 cells were elevated in the spleen, ILNs, brain, and spinal cord of constipation EAE mice and FMT EAE mice. Moreover, compared with EAE mice, the Treg/Th17 and Treg17/Teff17 ratio in these tissues were lower in the constipation EAE mice and FMT EAE mice (Figs. 4, 5).

### Constipation induced gut microbiota dysbiosis aggravated the cytokines disturbance in EAE mice

**Fig. 6.**
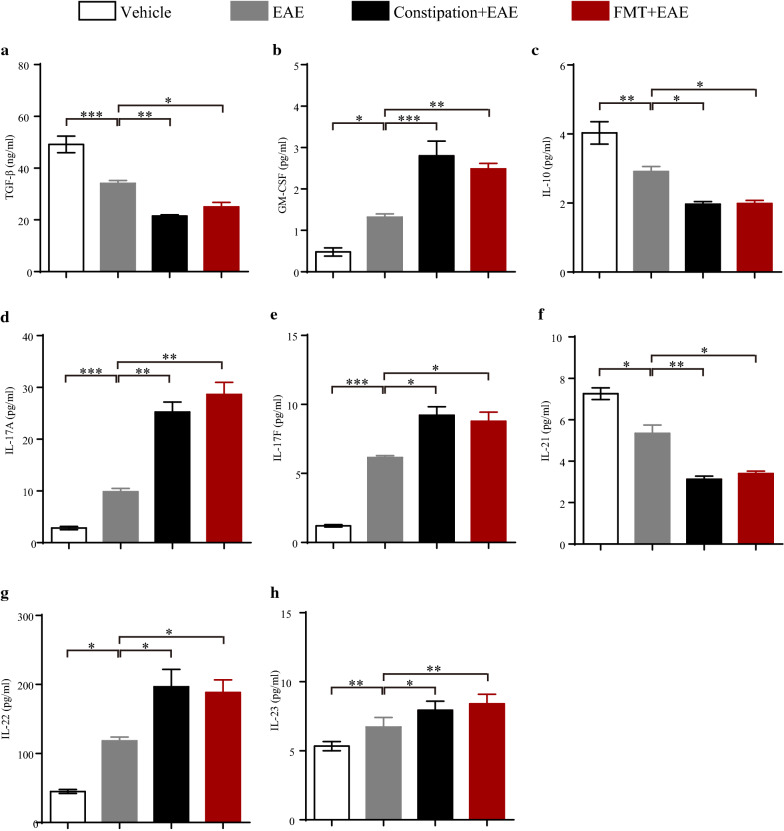
Constipation induced gut microbiota dysbiosis aggravated the cytokines disturbance in EAE mice. Serum samples of different treated mice were aseptically harvested on 25st day p.i. for circulating cytokines measurement. The level of TGF-β was determined using an ELISA Kit (**a**). The levels of GM-CSF (**b**), IL-10 (**c**), IL-17A (**d**), IL-17F (**e**), IL-21 (**f**), IL-22 (**g**) and IL-23 (**h**) were determined using a ProcartaPlex™ multiplex immunoassay. N = 6 in each group. Data are displayed as mean ± SEM. *p < 0.05, **p < 0.01, ***p < 0.001

TGF-β, IL-10, and IL-21 are Treg17 cells-associated cytokines, while GM-CSF, IL-17A, IL-17F, IL-22, and IL-23 are Teff17 cells-associated cytokines [18, 33–35]. Serum was harvested at 25st days p.i. from each group for these circulating cytokines measurement. We found that the concentrations of TGF-β, IL-10, and IL-21 were decreased while the levels of GM-CSF, IL-17A, IL-17F, IL-22, and IL-23 were increased in constipation EAE mice and FMT EAE mice compared to EAE mice (Fig. 6).

## Discussion

Constipation is one of the commonest symptoms associated with stressful lifestyle [3–5]. Here, we demonstrated that constipation induced gut microbiota dysbiosis, colon injury, and increased permeability of intestinal barrier and BBB in C57BL/6 mice. In the typical animal model of MS, constipation exacerbated EAE through aggravating Treg/Th17 and Treg17/Teff17 imbalance and cytokines disturbance. And these pathological processes could be transferred via their gut microbiota.

We found that constipation mice exhibited significantly reduced abundance and diversity of gut microbiota with elevated F/B ratio in the phylum level. The increased F/B ratio have been tightly linked to the pro-inflammatory environment and immunological imbalance characteristic of autoimmune disorders [36, 37]. And it has been reported to be associated with elevated Th17 cells in the intestine and the disease activity of MS patients [38–40]. Moreover, we found that the abundance of *Prevotella*, *Ruminococcus* and *Turicibacter* were increased while *Lactobacillus* was decreased in constipation mice. *Prevotella* could activate Toll-like receptor 2, promoting the secretion of Th17-polarizing cytokines such as IL-23 and IL-6. *Turicibacter* was considered the pro-inflammatory taxa in chronic inflammatory disease [41, 42]. *Ruminococcus* promoted the Th17 cells differentiation and disrupted the Treg/Th17 balance, while *Lactobacillus* exerted opposite function [43]. We inferred that the alterations in these specific gut microbiotas may indicate the disturbed intestinal immune state in the constipation mice. And it was further verified by the increased inflammation and injury of colon that we observed in the constipation mice.

We also found that the permeability of intestinal barrier in constipation mice was increased. The intestinal barrier limits the host’s contact with noxious luminal antigens, while a barrier defect has been involved in the pathogenesis of autoimmune diseases such as inflammatory bowel disease (IBD), celiac disease, irritable bowel syndrome (IBS), type 1 diabetes mellitus, and MS [44–48]. Constipation induced microbiota dysbiosis can lead to the increased intestinal permeability in mice, which is supported by previous studies [1, 7]. A dysfunctional intestinal barrier could permit microbiota driven proinflammatory state with impacts on the CNS [26], which was supported by the increased BBB permeability of the constipation mice that we observed. BBB acts as a gatekeeper in CNS, protecting the brain from exposure to antigens that may be harmful to neurons [49]. It has been reported that BBB disruption could be caused by gut microbiota dysbiosis, leading to the pathogenic antigen and serum protein leakage into CNS. This process has been previously demonstrated in the studies of schizophrenia, depression, Alzheimer’s disease (AD), Parkinson’s disease (PD) and MS, although the exact route is unclear [39, 47, 49–52].

Defects of the permeability of intestinal and BBB in constipation mice could influence the host immune functions, so we verified the effects of constipation on EAE mice. We found that constipation exacerbated EAE whether evaluated by clinical or pathological scores. And constipation aggravated the Treg/Th17 and Treg17/Teff17 imbalance both in the peripheral lymphoid organs and CNS in EAE mice. Treg cells and Th17 cells are two particular T cell subsets with plasticity, and their differentiations are influenced by the surrounding microenvironment [13–15]. Moreover, the Treg/Th17 balance plays an essential role in the pathogenesis of MS and EAE [16]. Th17 cells display remarkable heterogeneity and plasticity depending on different microenvironment [13–15]. Treg17 cells are novelly defined anti-inflammatory mediator, while Teff17 cells are important pathogenic cells [18, 19]. Moreover, the Treg17/Teff17 imbalance has been implicated in a series of autoimmune diseases including giant cell arteritis, lupus nephritis, and glomerulonephritis, which supports that Treg17/Teff17 imbalance acts as a feature of autoimmune diseases [19, 20, 53]. Here we found the imbalance of Treg/Th17 and Treg17/Teff17 in EAE mice, and constipation aggravated the Treg/Th17 and Treg17/Teff17 imbalance in EAE mice. As T cells abundantly reside in the intestinal lamina propria, constipation induced microbiota dysbiosis and the increased permeability of intestinal barrier and BBB may contribute to the increased pathological T cells into blood circulation and CNS then aggravated Treg/Th17 and Treg17/Teff17 imbalance in the constipation EAE mice.

Our findings are also supported by preclinical evidences that constipation induced microbiota dysbiosis could modulate brain functions by immune, endocrine and neural pathways through the brain-gut-microbiota axis in other diseases [54]. It has been reported that constipation induced microbiota dysbiosis and damaged barrier function in the pathogenesis of depression, AD and PD [55–57]. Although the detailed mechanistic insights of these interactions are currently underdeveloped, it is considered that constipation induced “leaky” intestinal barrier and BBB may enable the interactions among gut microbiota, immune system, and brain through multidirectional pathways [49]. FMT from the diseased animal donors to microbiota-depleted mice is the conventional manipulation for the reestablishment of gut microbial community, which could passively induce the pathological process of the diseases [58]. In this study, we found that FMT from constipation mice could exacerbate clinical severity, and Treg/Th17 and Treg17/Teff17 imbalance in EAE mice as constipation did in EAE mice. It further suggests the effects of constipation induced microbiota dysbiosis on EAE mice.

Circulation cytokines are important mediators in the communication between intestine and brain [49]. Moreover, they are well-known as the key players in the pathogenesis of MS and EAE [59]. We found the reduced levels of TGF-β, IL-10, and IL-21, and the increased levels of GM-CSF, IL-17A, IL-17F, IL-22, and IL-23 in the serum of constipation EAE mice. TGF-β, IL-10, and IL-21 were identified as typical anti-inflammatory cytokines reducing T cells trafficking and T cells responses in EAE [60, 61]. GM-CSF, IL-17A, IL-17F, IL-22 and IL-23 are Th17 cell-related cytokines, which were associated with the aggravation of immune response in EAE [62, 63]. Th17 and Teff17 cells promote the secretions of GM-CSF, IL-17A, IL-17F and IL-22, while Treg and Treg17 cells promote the production of IL-10 and IL-21 [18]. The disturbance of circulation cytokines was consistent with the imbalance of Treg/Th17 and Treg17/Teff17 cells in the peripheral immune system and CNS in the constipation EAE mice. That whether the disturbance of circulation cytokines affected Treg/Th17 and Treg17/Teff17 cells imbalance or the imbalance of Treg/Th17 and Treg17/Teff17 cells influenced the circulation cytokines disturbance remains unknown, but the gut dysbiosis and its influence on host barrier function may be a critical node of the changes of immune microenvironment [64]. Our results provides evidences for the assumption that the complex interplay of constipation induced microbiota dysbiosis in the changed immune microenvironment in EAE. Similarly, FMT from constipation mice in EAE could produce these effects in our study.

In summary, our study demonstrated that constipation induced gut microbiota dysbiosis and increased permeability of intestinal barrier and BBB, which contributes to the aggravation of clinical features, the Treg/Th17 and Treg17/Teff17 imbalance, and the cytokines disturbance in EAE mice. The findings may help to further understand the effects of constipation induced gut microbiota dysbiosis in the pathogenesis of EAE. Since constipation is one of the most important stressful lifestyle related symptoms, this study implies the potential of lifestyle modulation in the alleviation of MS.

## Supplementary Information

**Additional file 1:** **Fig. S1. **Antibiotic cocktail treatment significantly depleted the gut microbiota in C57BL/6 mice. Four- to five -week-old female C57BL/6 mice were administrated with antibiotic cocktail for 2 weeks. Fresh extruded stools were collected right after the antibiotic cocktail treatment for 16S rRNA sequencing analysis. The indexes of observed species, Chao, Ace, Shannon and Simpson in the vehicle mice and microbiota-depleted mice (**a**). PLS-DA analysis based on OTU abundance information (**b**). Relative abundances of the gut microbiota at phylum level (**c**) and genus level (**d**). Cladogram using LEfSe analysis indicated the phylogenetic distribution of gut microbiota (**e**). LDA scores showed the significant bacterial differences between the vehicle mice and microbiota-depleted mice (**f**). N = 6 in vehicle group, and N = 8 in microbiota-depleted group. Data are displayed as mean ± SEM. *p < 0.05, **p < 0.01, ***p < 0.001.

## Data Availability

The datasets used and/or analyzed during the current study are available from the corresponding author on reasonable request.

## Abbreviations

EAE: Experimental autoimmune encephalomyelitis
MS: Multiple sclerosis
FMT: Fecal microbiota transplantation
CNS: Central nervous system
Treg: Regulatory T
Th: T helper
IL: Interleukin
TGF-β: Transforming growth factor β
Treg17: Regulatory Th17
Teff17: Effector Th17
BaSO_4_: Barium sulfate
FITC-D4000: Fluorescein isothiocyanate-dextran 4000
EB: Evans blue
CFA: Complete Freund’s adjuvant
MOG: Myelin oligodendrocyte glycoprotein
PTX: Pertussis toxin
ELISA: Enzyme-linked immunosorbent assay
GM-CSF: Granulocyte-macrophage colony-stimulating factor
PBS: Phosphate-buffered saline
PLS-DA: Partial least squares discrimination analysis
OUT: Operational taxonomic units
LEfSe: Linear discriminant analysis effect size
PFA: Paraformaldehyde
TEM: Transmission electron microscopy
BBB: Blood–brain barrier
HE: Hematoxylin and eosin
LFB: Luxol fast blue
ILNs: Inguinal lymph nodes
SEM: Standard error of the mean
F/B ratio: *Firmicutes*/*Bacteroidetes* Ratio
IBD: Inflammatory bowel disease
IBS: Irritable bowel syndrome
AD: Alzheimer’s disease
PD: Parkinson’s disease
